# Topical recombinant human Nerve growth factor (rh-NGF) is neuroprotective to retinal ganglion cells by targeting secondary degeneration

**DOI:** 10.1038/s41598-020-60427-2

**Published:** 2020-02-25

**Authors:** Li Guo, Benjamin M. Davis, Nivedita Ravindran, Joana Galvao, Neel Kapoor, Nasrin Haamedi, Ehtesham Shamsher, Vy Luong, Elena Fico, M. Francesca Cordeiro

**Affiliations:** 1 Glaucoma & Retinal Neurodegeneration Research Group, Institute of Ophthalmology, University College London, London, United Kingdom; 2 Department of Biotechnological and Applied Clinical Sciences, University of L’Aquila, L’Aquila, Italy; 3 Western Eye Hospital, Imperial College Healthcare NHS Trust, London, United Kingdom

**Keywords:** Cell death in the nervous system, Translational research

## Abstract

Optic neuropathy is a major cause of irreversible blindness worldwide, and no effective treatment is currently available. Secondary degeneration is believed to be the major contributor to retinal ganglion cell (RGC) death, the endpoint of optic neuropathy. Partial optic nerve transection (pONT) is an established model of optic neuropathy. Although the mechanisms of primary and secondary degeneration have been delineated in this model, until now how this is influenced by therapy is not well-understood. In this article, we describe a clinically translatable topical, neuroprotective treatment (recombinant human nerve growth factor, rh-NGF) predominantly targeting secondary degeneration in a pONT rat model. Topical application of rh-NGF twice daily for 3 weeks significantly improves RGC survival as shown by reduced RGC apoptosis *in vivo* and increased RGC population in the inferior retina, which is predominantly affected in this model by secondary degeneration. Topical rh-NGF also promotes greater axonal survival and inhibits astrocyte activity in the optic nerve. Collectively, these results suggest that topical rh-NGF exhibits neuroprotective effects on retinal neurons via influencing secondary degeneration process. As topical rh-NGF is already involved in early clinical trials, this highlights its potential in multiple indications in patients, including those affected by glaucomatous optic neuropathy.

## Introduction

Secondary degeneration occurs commonly in the central (CNS) and peripheral (PNS) nervous systems, where injury from initial lesions can lead to widespread damage to neurons far beyond the primary injury site^1^. The second phase of injury is thought to be caused by the accumulation of noxious factors such as oxidative radicals, glutamate and calcium release from primarily damaged cells and peripheral immune cell activation^2–5^. Secondary neurodegeneration is believed to be the major contributor to neuronal death in CNS injuries including those affecting the spinal cord^1,6^ and in optic neuropathies, such as glaucoma, ischaemic optic neuropathy, and Leber’s hereditary optic neuropathy^7–9^.

Optic neuropathy describes a collection of disorders characterised by damage to the optic nerve and loss of retinal ganglion cells (RGCs) due to any cause, including glaucoma, ischaemia, trauma and genetic predisposition^10,11^. Of these, glaucoma represents the leading cause of global irreversible blindness, affecting over 60.5 million people, a figure set to double by 2040^9,12^. Currently, intraocular pressure (IOP) presents the only therapeutically modifiable risk factor for glaucoma^13^; however, patients with well-controlled IOP can still lose vision (disease progression), necessitating the development of novel, non-IOP-dependent treatment strategies for this condition^14^. Neuroprotective therapies are increasingly recognized as a promising approach to slow or prevent optic neuropathy associated RGC loss^15,16^, and the use of new endpoints and biomarkers, such as DARC (Detection of Apoptotic Retinal Cells)^17^, may significantly improve their assessment and shorten their clinical development time.

Neurotrophins are a family of proteins that regulate the growth and survival of nerve cells. Nerve growth factor (NGF) is an endogenous secreted neurotrophic factor that has been widely investigated as a promising neuroprotective agent since its discovery in 1950^18^. NGF is thought to act through interaction with the transmembrane tyrosine kinase receptor (TrkA) and p75 neurotrophin receptor (p75NTR)^19,20^. NGF and its receptors are highly expressed throughout the visual system, from the retina to the visual cortex^21^. In the retina, RGCs have been shown not only to express NGF and its receptors, but also to transport NGF in a retrograde and anterograde fashion along their axons, suggesting the role of NGF in maintenance of RGC function^22^. Expression profiles of endogenous NGF and its receptors are found to be altered in the retina and optic nerve in experimental glaucoma associated with RGC loss^23–25^. Topical NGF application has previously been shown to be neuroprotective in glaucoma, with evidence of reduced RGC loss in experimental models and a suggestion of improved visual function in patients^25,26^. Pharmacokinetic analysis has confirmed that single topical dose of ^125^I-labeled NGF enabled penetrating the eye and reaching the retina and optic nerve^27^.

To establish whether the neuroprotective effects of topical NGF occur via primary or secondary neurodegenerative processes, this study investigates the effect of this therapeutic intervention on patterns of RGC loss in a partial optic nerve transection (pONT) model^7,28,29^. The pONT is a well-established animal model, induced by transecting only the dorsal axons of the optic nerve, leaving ventral axons intact but vulnerable to secondary degeneration^25,28^. Using this model, we have previously successfully quantified the components of primary and secondary degeneration and evaluated therapeutic strategies^30,31^.

In the present study, we used the same model to assess the effects of topical recombinant human NGF (rh-NGF) on RGC protection by assessing both RGC apoptosis *in vivo* with DARC and RGC survival histologically. Recently established Brn3a^+^ whole-retinal RGC soma counting algorithms were applied to evaluate the effect of NGF mediated neuroprotection on primary and secondary neurodegenerative processes^30,32,33^. Finally, to evaluate its effects on RGC axons, we assessed RGC axonal health and astrocytic activity.

## Results

### Topical rh-NGF significantly reduced pONT-induced RGC apoptosis *in vivo*

**Figure 1:**
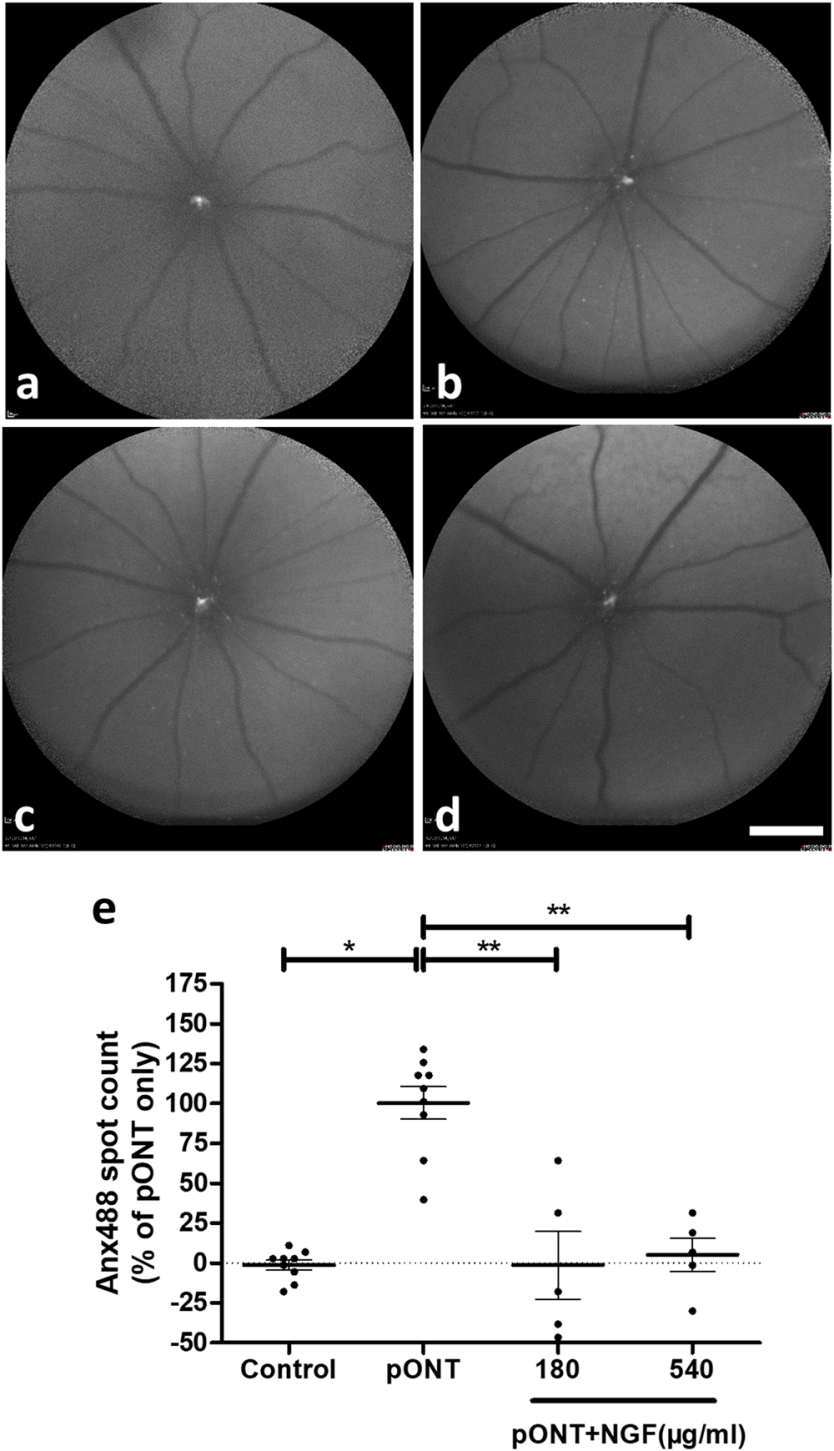
Topical rh-NGF significantly reduced pONT-induced RGC apoptosis *in vivo*. DARC images show RGC apoptosis (white spots) in naïve control (**a**), pONT only (**b**), pONT + NGF (180 µg/ml, **c**) and pONT + NGF (540 µg/ml, **d**). By normalizing RGC apoptosis counts as percentage to pONT only, significant protective effects of rh-NGF were found with both 180 µg/ml and 540 µg/ml doses (**e**). Results are means ± SEM. All stats are one-way ANOVA with Bonferoni post-test, *p < 0.05, **p < 0.01. N ≥ 5. Scale bar: 1 mm.

The effects of topical rh-NGF on RGC protection were firstly assessed *in vivo* using DARC (Detection of Apoptosing Retinal Cells)^17^ imaging. Increased levels of RGC apoptosis were seen after pONT induction compared to naïve controls; however, topical rh-NGF treatment significantly reduced apoptosis, as seen in Fig. 1a–e. Results are shown after normalizing RGC apoptosis counts to those in pONT alone, with significant (p < 0.01) protective effects of rh-NGF found with both 180 µg/ml and 540 µg/ml doses to a level comparable to naïve controls (see Eq. 1 in the Methods).

### Topical rh-NGF significantly promoted RGC soma survival

To corroborate our *in vivo* observations, the neuroprotective effects of topical rh-NGF administration were next assessed histologically by counting the number of Brn3a^+^ RGC soma in retinal whole-mounts using previously well-established techniques^31^. Brn3a is a nuclear restricted transcription factor that has previously been reported to label 97% of the rodent RGCs (excluding melanopsin expressing RGCs) and this signal is rapidly lost by RGCs undergoing cell death processes^33^. RGC survival in each retinal whole-mount was assessed using three previously established parameters: mean RGC density (RGC/mm^2^), nearest neighbour distance (NND) and regularity index (RI). NND refers to the distance between each neuron and its nearest neighbour in the mosaic, presented as the mean NND. RI is a measure of RGC mosaic regularity calculated by dividing mean NND by the standard deviation of the NND of a population^34^.

**Figure 2:**
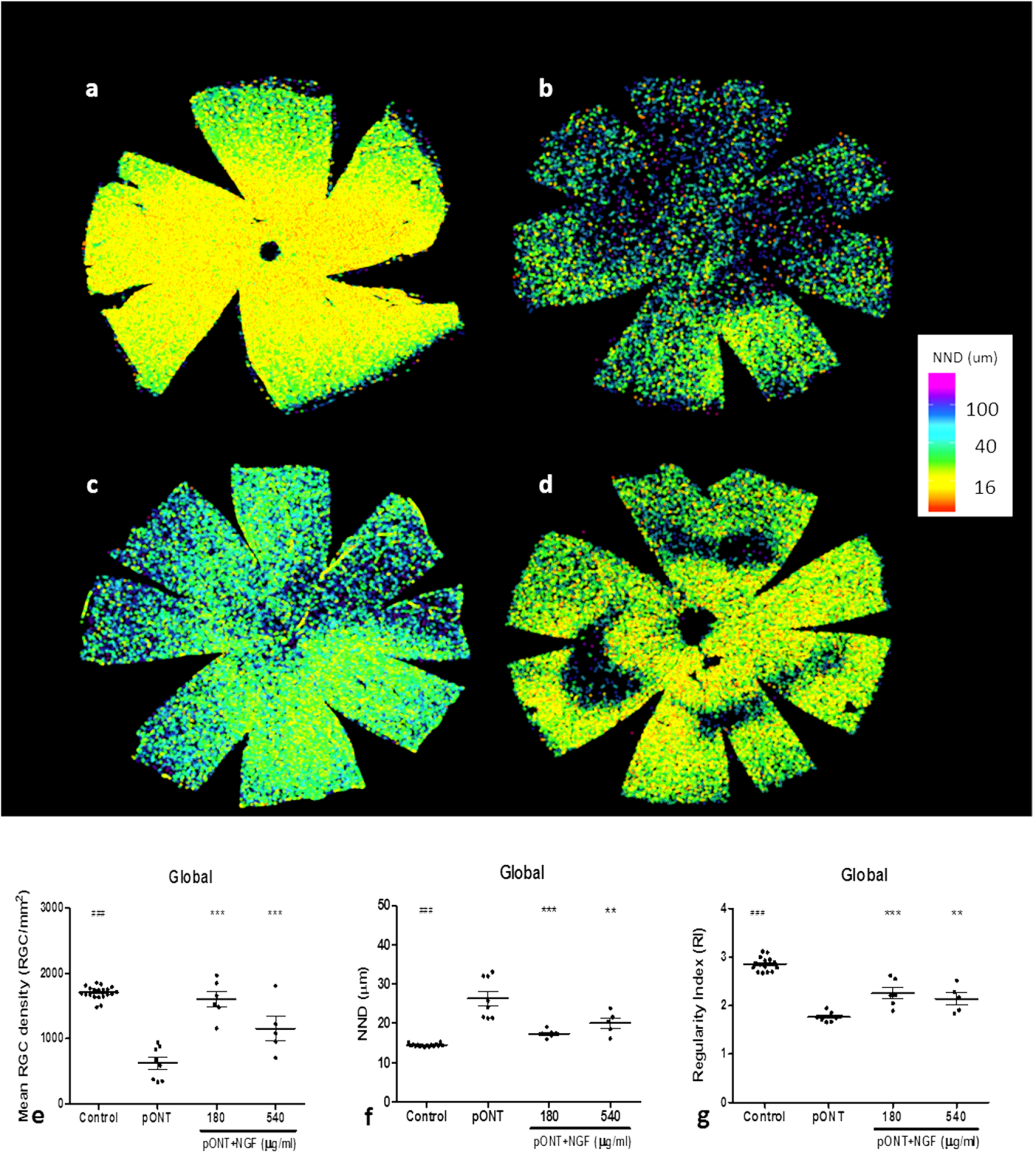
Topical rh-NGF promoted RGC soma survival in the retinal whole-mounts. (**a**–**d**) Brn-3a labelled RGC distribution maps in Normal retina (**a**), pONT only (**b**), pONT + NGF 180 µg/ml (**C**), and pONT + NGF 540 µg/ml (**d**). The protective effects of topical rh-NGF on Brn3a+ cell density (cells/mm^2^) (**e**), nearest neighbour distance (NND) (**f**) and Regularity Index (RI) (**g**). Topical rh-NGF in both doses (180 and 540 µg/ml) significantly promoted RGC survival by enhancing cell density, reducing NND, and remaining RI. pONT induced a significant damage of RGCs in the three parameters (RGC density, NND, and RI), compared to normal control. Results are means ± SEM. All stats are one-way ANOVA with Bonferoni post-test, ^###^p < 0.001: normal control compared to pONT; *p < 0.05, **p < 0.01, ***p < 0.001: NGF treatments compared to pONT. N ≥ 5. Scale bar: 2 mm.

The whole-mount retinal images showed that Brn-3a^+^ RGC density was substantially reduced in pONT only (without NGF treatment), compared to naïve control, while less reduction of RGC density was seen in retinas treated by topical rh-NGF (Fig. 2a–d). pONT resulted in a significant reduction in RGC density and regularity index, and a significant increase in mean nearest neighbour distance (Fig. 2e–g). Topical rh-NGF in both doses significantly reduced pONT-induced RGC damage, by increasing mean RGC density and preserving NND and regularity of RGCs. Between them, topical rh-NGF in 180 µg/ml dosing appeared to be most effective in preserving cell survival, reaching to significance in stabilizing cell regularity. These results support the above *in vivo* observations that topical rh-NGF reduces RGC apoptosis in the pONT model of optic nerve injury.

### Topical rh-NGF predominantly prevented secondary RGC degeneration

**Figure 3:**
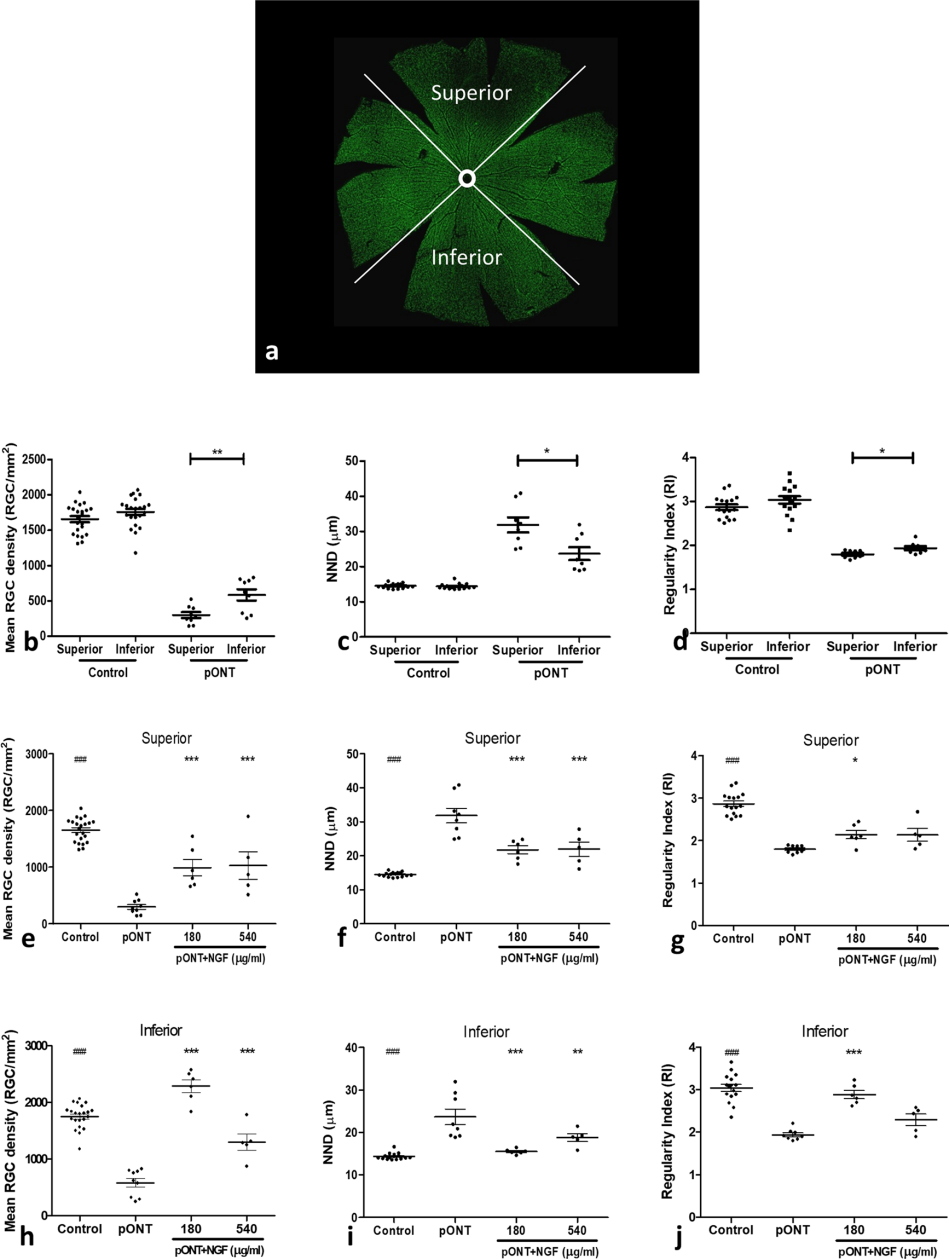
Topical rh-NGF predominantly prevented secondary RGC degeneration. Segmentation of retinal whole-mount into superior and inferior quadrants (**a**). Comparison of pONT induced damage in the superior and inferior retinas (**b**–**d**). The effects of topical rh-NGF, in the superior (**e**–**g**) and Inferior (**h**–**j**) retina, on Brn3a+ RGC density (**e**,**h**), nearest neighbour distance (NND) (F and I) and Regulatory index (RI) (**g**,**j**). Topical rh-NGF significantly protected RGCs in both superior and inferior retina, but more effective in the inferior with lower dosed NGF. Results are means ± SEM. All stats are one-way ANOVA with Bonferoni post-test, ^###^p < 0.001: normal control compared to pONT; *p < 0.05, **p < 0.01, ***p < 0.001: NGF treatments compared to pONT. N ≥ 5.

Having established that topical rh-NGF reduced RGC apoptosis and promoted RGC soma survival after pONT, we next determined whether the neuroprotective effects of rh-NGF were mediated by effects on primary or secondary degenerative processes. This was achieved by dividing each retinal whole-mount into superior and inferior quadrants as previously described^30,31^ (Fig. 3a), where only dorsal optic nerve axons are primarily injured (cut) corresponding to the superior retina, while ventral axons remain intact but vulnerable to secondary degeneration^31^. Our results in this study show greater loss of RGCs in the superior retinal quadrant following initial pONT induction (superior cut) (p < 0.01), accompanied by increased nearest neighbour distance (p < 0.05), and reduced regularity index (p < 0.05), respectively (Fig. 2b–d).

Topical rh-NGF administration exhibited significantly protective effects in both superior and inferior retina quadrants (Fig. 3e–j). However, a greater proportion of the RGC soma population was preserved in the inferior (Fig. 3h–j) than superior (Fig. 3e–g) retinal quadrants, where mean RGC density in the inferior retina was comparable to naïve controls. Similarly, a greater preservation in both nearest neighbour distance and regularity index was evidenced in the inferior than superior quadrants. In addition, topical rh-NGF had almost no significant effect in the superior retina on stabilizing cell regularity although mean RGC density and NND were protected. This suggests that topical rh-NGF administration predominantly protects RGCs against secondary neurodegenerative processes which accounts for a greater proportion of RGC loss in the inferior retinal quadrant.

### Topical rh-NGF promoted RGC axonal survival and reduced glial activation

**Figure 4:**
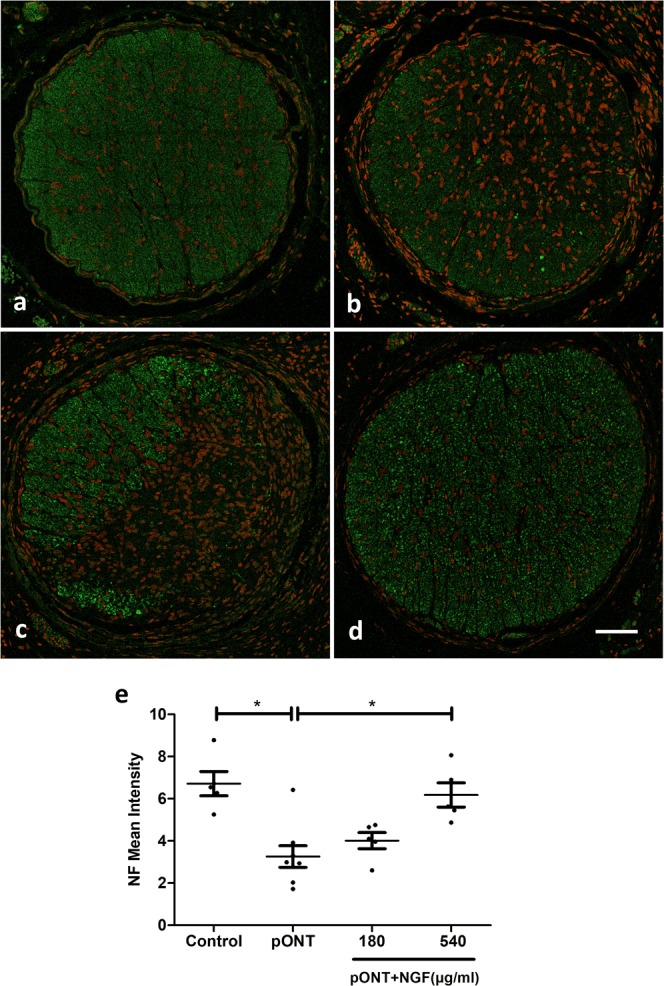
Topical rh-NGF promoted optic nerve axonal survival. Confocal images showed NF immunostaining (green) of the optic nerve in treatment groups of naïve (**a**), pONT only (**b**), pONT + NGF 180 µg/ml (**c**), and pONT +NGF 540 µg/ml (**d**). (**e**) Data analysis showed that pONT induced a significant reduction in NF-stained axonal intensity (pONT only) compared to naïve group (p < 0.05). Topical rh-NGF (540 µg/ml) treatment significantly enhanced axonal intensity compared to pONT only group (p < 0.05). However, lower rh-NGF (180 mg/ml) did not show significant protection. DAPI staining of nuclei in red. Results are means ± SEM. All stats are one-way ANOVA with Bonferoni post-test, *p < 0.05. N ≥ 5. Scale bar: 0.1 mm.

Having determined that topical rh-NGF was significantly effective in protection of RGC soma and reducing levels of retinal apoptosis *in vivo* following pONT, we next sought to investigate whether the rh-NGF therapies could preserve RGC axons in the optic nerve. This was achieved using immunofluorescence techniques with antibodies of anti-mouse neurofilament-H (NF) and anti-rabbit glial fibrillary acidic protein (GFAP) to assess axonal survival and astrocyte activity, respectively. pONT induction was found to result in significant reduction of the intensity of NF labelled optic nerve axons, compared to naïve controls (p < 0.05, Fig. 4a,b). Topical rh-NGF administration was found to result in a dose dependent increase in NF fluorescence, achieving significance at the higher (540 µg/ml, p < 0.05), but not lower (180 µg/ml, p > 0.05) doses (Fig. 4c,d). These results suggest that topical rh-NGF promotes greater axonal survival and/or up-regulation of NF, which is a pro-axon survival factor^35^.

**Figure 5:**
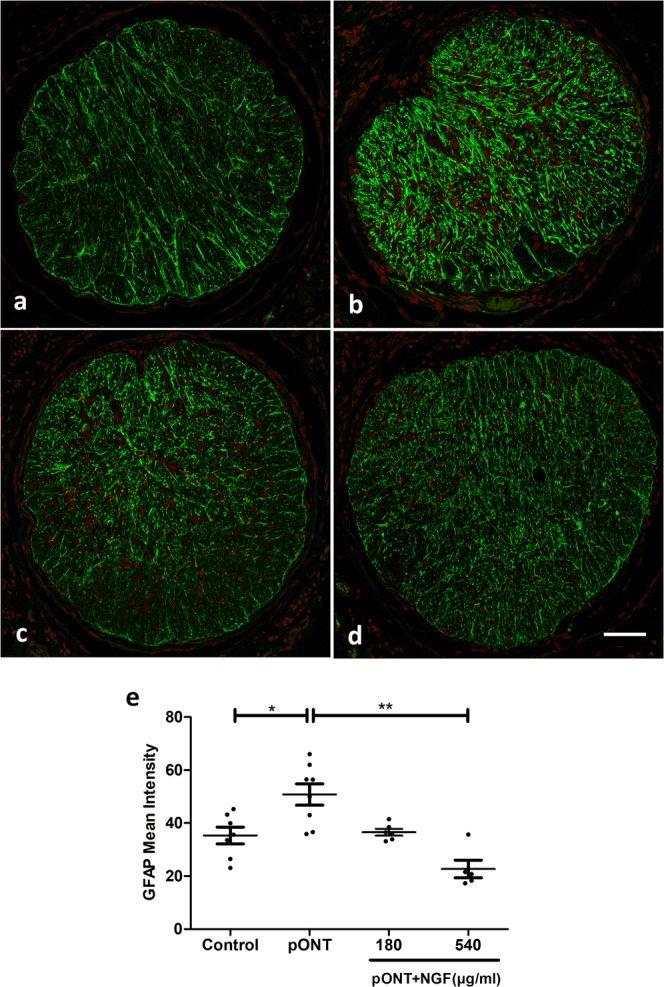
Topical hr-NGF inhibited glial cell activity. Confocal images showed GFAP immunostaining of the optic nerve in treatment groups of naïve (**a**), pONT only (**b**), pONT + NGF 180 µg/ml (**c**), and pONT + NGF 540 µg/ml (**d**). (**e**) pONT induced a significant increase in GFAP intensity compared to naïve controls, topical rh-NGF (540 µg/ml) treatment significantly reduced GFAP intensity, suggesting inhibiting of astrocyte activity (p < 0.01). Lower dose of rh-NGF (180 µg/ml) therapy showed a trend of reduction, but not reaching significance. Results are means ± SEM. All stats are one-way ANOVA with Bonferoni post-test, *p < 0.05, **p < 0.01. N ≥ 5. Scale bar: 0.1 mm.

GFAP immunostaining revealed a significant increase in astrocyte activity following pONT induction (p < 0.05, Fig. 5), which was significantly prevented by higher dose of rh-NGF (540 µg/ml) (p < 0.01, Fig. 5), suggesting that NGF may attenuate astrocyte proliferation.

Together these data suggest that while the lower doses of topically applied rh-NGF can preserve RGC soma function, higher doses are required to have beneficial effects on optic nerve axons and astrocyte health.

### rh-NGF reached the optic nerve after topical administration

**Figure 6:**
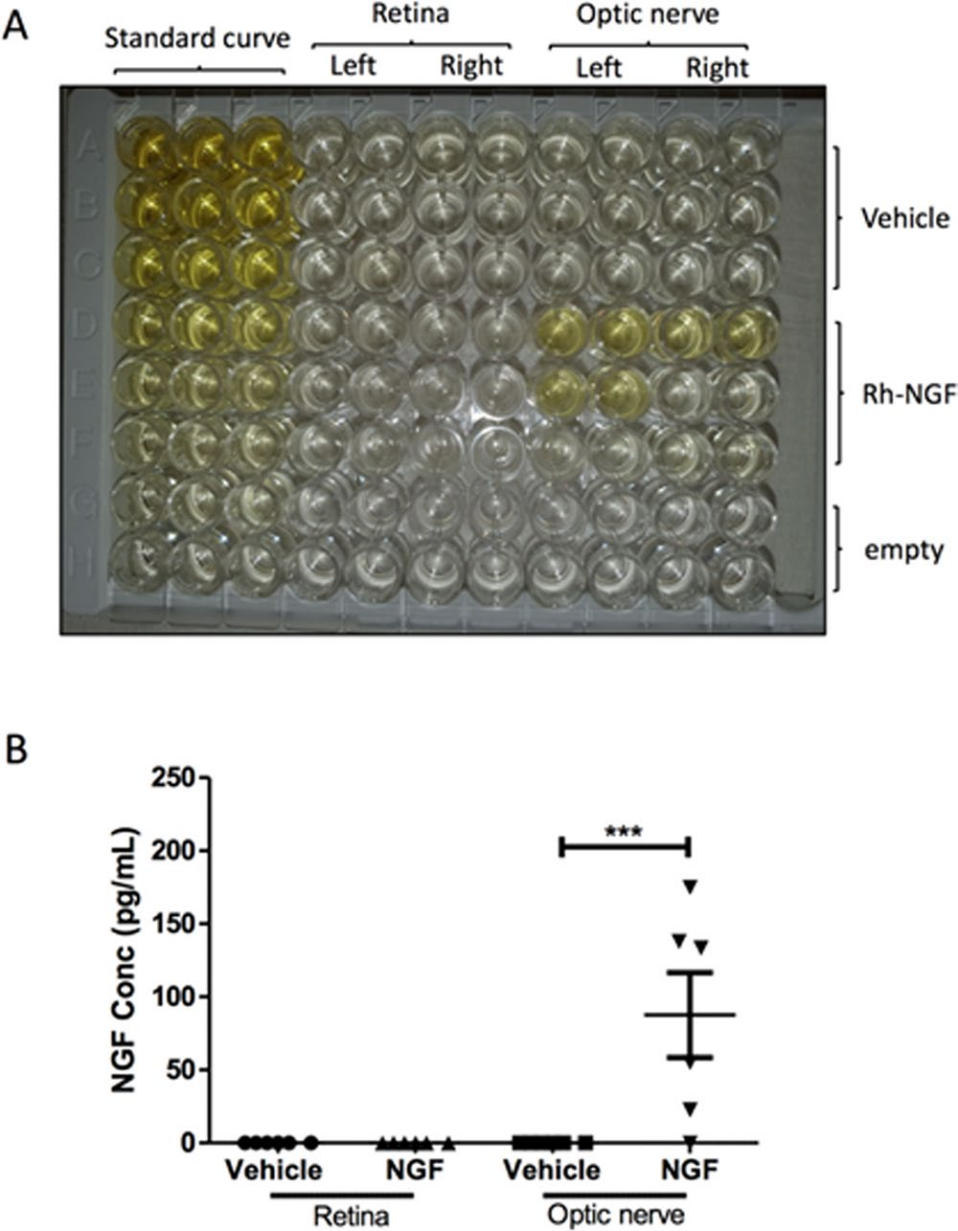
rh-NGF reached the optic nerve after topical administration. ELISA assay results of ocular tissues showed that rh-NGF was present in the optic nerve following topical administration (**A**, yellow) but not in the retina. Compared to vehicle, topical rh-NGF treatment resulted in a significant level of rh-NGF in the optic nerve (p < 0.001, **B**). There was no significant difference of rh-NGF levels in the optic nerve between the left eyes (with pONT) and the right eyes. Results are means ± SEM. All stats are one-way ANOVA with Bonferoni post-test, ***p < 0.001.

Having demonstrated that topical rh-NGF was neuroprotective to RGCs and their axons, we next looked at whether rh-NGF could be detected in the retina and optic nerve after topical administration by ELISA assay. Whereas rh-NGF was absent in vehicle treated eyes, it was detected in the optic nerve with a mean concentration of 87.52 pg/ml (p < 0.001, Fig. 6), though not present in the retina. There was no significant difference of rh-NGF levels in the optic nerve between the left eyes (with pONT) and the right eyes.

## Discussion

This study demonstrates that topical rh-NGF is neuroprotective to RGCs and their axons in an optic neuropathy model of pONT. Our results suggest that topical rh-NGF therapy significantly reduces RGC apoptosis *in vivo* using DARC and promotes RGC soma survival using whole-retinal Brn3a+ histological assessments. The neuroprotective effects of rh-NGF are likely associated with preventing secondary neurodegenerative processes. Topical rh-NGF also enhances RGC axonal survival and reduces astrocytic activity in the optic nerve after pONT.

Although optic neuropathy related RGC death can lead to profound vision loss, efficient treatment is currently not comprehensive. However, since the first evidence for a role of NGF in CNS development and regeneration in the late 1970’s, the prospect of nerve growth factor mediated nerve protection has been recognized^36–38^. Previously, it has been shown that intravitreal administration of exogenous NGF exhibits neuroprotective effects by increasing RGC survival seen in various animal models of optic neuropathy, including ON axotomy^36,38,39^, OHT glaucoma^20,25,26,40,41^, and diabetic retinopathy^40,42^. While repeated intraocular injections of exogenous NGF could enhance RGC survival^38,39^, its administration could have drawbacks, such as discomfort for patients, expensive for hospitals and potential vision threatening risks^43^. Topical administration of NGF therefore has been attempted and demonstrated to be effective experimentally^25,26,40,42^. Topical NGF is already approved for treatment in humans with neurotrophic keratitis^44^, and is safe, well tolerated and effective in patients^26,44–47^. It has not been used for any other clinical ocular indication.

In optic nerve disease, previous NGF studies have employed either retinal cross-sectioning to quantify surviving RGCs with non-specific staining, such as hematoxylin and eosin staining^25,26,36,42^, or sampling of retinal whole-mounts^38,39^. Although sampling RGC population from whole-mounts is more reliable than cross-sectioning counting, there remains considerable variability due to both human error and variations in RGC density across the retina, even when a large fraction of the retinal area (quadrant- or hemi-retina) is sampled^48^. To address the concerns of limitation in those approaches, we used Brn3a to label RGCs in retinal whole-mounts, and quantified all surviving RGCs by our novel algorithm^30–32^. Using this algorithm, information regarding the spatial distribution of RGCs, i.e. their spatial relationship and regularity, provides additional and valuable data to RGC density^31,32^. Furthermore, by segmenting the retina and monitoring RGC loss longitudinally, the extent of primary and secondary degeneration can be estimated.

Our results showed that topical rh-NGF was indeed significantly effective in retaining RGC survival 3 weeks after partial damage of the optic nerve, evidenced by increased RGC density and regularity, and decreased average nearest neighbour distance measures. Choosing 3 weeks to evaluate topical NGF was based on our previous findings that RGC loss in the pONT model has reached a maximum level at this time point^30–32^. By segmentation of the whole retina into quadrants, we further examined whether the protective effects of NGF were attributed to targeting primary and/or secondary degeneration. We found that although RGC density in the superior quarter was significantly higher in NGF treated groups than untreated group, the most pronounced improvement was identified in the inferior retina, where RGC survival in NGF treated groups was considerably comparable to that in naïve controls, which is indicative of secondary degeneration as the main target for NGF. This property of NGF would be extremely valuable for clinical application because secondary degeneration is the major concern in optic neuropathies, including normal-tension glaucoma where RGCs continue to die even at low intraocular pressures^43^. Perhaps counter-intuitively, the protective effect of NGF appeared to be more significant with a lower (180 µ/mg) than higher dose (540 µg/ml), in the inferior retina (p < 0.001). A possible explanation for this could be that more NGF may have been delivered to the retina with lower dosing concentrations, as supported by similar results in a previous study where a greater level of exogenous ^125^I-labeled NGF was transported into the retina after topically administrating of NGF at 200 µg/ml rather than 500 µg/ml^27^. In terms of pharmacokinetics, ^125^I-labeled NGF was detected in retinal ganglion cells and the optic nerve 2 hours after topical administration, followed by a peak level occurring at 6 hours but was undetectable after 48 hours^27^. This indicates that daily application of topical rh-NGF, described in the current study, would be at a sufficient level to provide neuroprotection after optic nerve injury.

Visual functional improvement following topical NGF treatment has been reported in patients, including three glaucoma patients assessed by pattern electroretinogram (PERG) and visual evoked potentials (VEPs)^26^, one patient with age-related macular degeneration (AMD) by visual acuity and focal ERG^49^, and five children with optic glioma and optic nerve atrophy by VEP^50^. In animal models, functional recovery has also been described following treatment with NGF^51,52^. Unfortunately, we were unable to perform visual functional assessment in the current study and it would be interesting to look into it in the future.

The mechanisms through which topical NGF is neuroprotective by mainly targeting secondary degeneration remain unclear. However, it could be associated with properties of NGF against the reactive metabolic events occurring in secondary degenerative processes, including apoptosis, oxidative stress, excitotoxicity, calcium dysregulation, and mitochondrial dysfunction^2–4^. NGF mediated prevention of RGC apoptosis has been documented by others previously^25,26^. NGF also exhibits neuroprotection by inhibiting oxidative stress via a mechanism involving induction of the phosphatidylinositol 3-kinase (PI3K)/Akt1 survival pathway^53^. Calcium dysregulation and excitotoxicity are the major events causing neuronal death in optic neuropathy, and have been implicated in secondary degeneration^28,54,55^. The neurotrophins, including NGF, play a key role in control of intracellular calcium levels and regulation of synaptic plasticity. NGF appeared to act on its target cells by inducing calcium influx and intracellular calcium mobilization^56^. The neurotrophin-mediated presynaptic uptake of calcium has been found to stimulate presynaptic release of neurotransmitter, a key element in synaptic plasticity and long term potentiation^56^. NGF is also crucial in maintenance of mitochondrial structure and function. A recent study has shown that NGF pre-treatment protects endothelial cells from indomethacin-induced injury through direct action on mitochondria via receptor TrkA, and the protective effect appeared to prevent mitochondrial membrane potential (MMP) depolarization^57^.

While RGC survival can be assessed and quantified histologically in animal models, imaging of RGCs *in vivo* has been challenging owing to the optical transparency of these cells^58^. However, as RGC loss in optic neuropathies have been shown to occur by apoptosis^11,59^, apoptotic RGCs can be imaged *in vivo* in real time using DARC technology that utilizes fluorescently labelled Annexin V to identify retinal neuronal apoptosis^60–62^. Established in 2004, the DARC has been demonstrated to be a valuable tool in monitoring of RGC apoptosis *in vivo* in various animal models of retinal neurodegeneration^62–66^. A recent clinical trial (Phase 1) in glaucoma patients has further shown that DARC enables visualization of neuronal apoptosis at single cell resolution in human retina, and DARC count (apoptosis) was significantly higher in glaucoma patients and correlated with disease progression^17^. Using DARC, we have shown in the present study that topical rh-NGF significantly reduced RGC apoptosis (Anx488 count) induced by pONT, compared to untreated controls. This is in agreement with previous studies that the protective effect of NGF on the RGCs is mediated by inhibition of apoptosis via increasing expression of pro-survival genes and decreasing expression of pro-apoptotic genes^25,26^. NGF-mediated reduction of apoptosis may also be associated with the restoration of the NGF/proNGF ratio, as proNGF protein is neurotoxic to RGCs and markedly up-regulated after axotomy^40,58,67^. The capability of imaging RGC apoptosis *in vivo* would pave the way to evaluate NGF therapy in patients with all kinds of optic neuropathy.

In addition to protection of RGC soma by enhancing RGC survival and reducing RGC apoptosis, we found that topical rh-NGF also protected RGC axons. The effects of NGF on the optic nerve appeared to be dose-dependent, which is not the case in RGC soma found in this study. The discrepancy could be explained by NGF reaching the retina and optic nerve via different routes. In support of the ‘different routes’ theory, a previous study has reported that topically administered NGF may reach the retina trans-sclerally, evidenced by increased levels in the sclera, and may reach the optic nerve via the retrobulbar space or systemic absorption^27^. Whereas the transscleral route has been seen on insulin uptake following topical administration^68^, delivery into the retrobulbar space to the optic nerve and retina is supported by other studies of topically instilled drugs, including iganidipine - a Ca^2+^ antagonist^69^ and Nepafenac – a non-steroidal anti-inflammatory agent^70^, in rabbits. The drugs were found to reach either the retrobulbar periocular space or the retina by local penetration at functional concentrations^69,70^. Additionally, a clinical study reported that a substantial amount of drugs was found to accumulate in the periocular tissue in glaucoma patients under long-term topical administration of timolol or betaxolol, suggesting that the topically instilled drugs exert pharmacologic effects by delivery into the retrobulbar space before reaching the posterior eye^71^. A similar finding has been described by the manufacture, Dompe, with topically administered rh-NGF; rh-NGF is found to be present in the retina and optic nerve but absent in both the aqueous humour and vitreous humour (Supplementary Fig. 1). Our ELISA assay results are consistent with this: rh-NGF is present in the optic nerve following topical rh-NGF administration but not in the retina, suggesting a retrobulbar route. We believe that reduction in RGC loss can be attributed to retrograde axonal protection of rh-NGF from the optic nerve. Alternatively, it may be due to accumulative effects of rh-NGF in the retina via the transscleral route following a twice-daily 21-day regimen, a similar observation to topical administration of iganidipine^69^. In addition to local penetration, systemic absorption has also been suggested by the findings of increased levels of NGF in the contralateral eye, including the retina, optic nerve and sclera, after topical unilateral administration, indicating that NGF has passed through the blood-ocular barrier^27^ though there is disagreement with topical iganidipine administration^69^.

Glial cell activation has been implicated in optic nerve injury. Activated astrocytes, as an early event after injury, have been documented to be a major contributor to spreading and acceleration of secondary degeneration^54,55^. Indeed, astrocytes undergo morphological changes in glaucomatous optic nerve, which precede the damage of RGC axons and somas^72,73^. Hypertrophy of astrocytes in glaucomatous optic nerve is related to early up-regulation of phosphorylated STAT3 (signal transducer and activator of transcription protein 3), a key transcription factor that regulates the expression of many genes and may play a crucial role in the initial activation of astrocytes in glaucoma^73^. It is thus proposed that glaucomatous damage in the optic nerve axons is secondary to astrocytes-induced damage^74^.

An important function of astrocytes is to preserve integrity of neural tissues following injury or in disease. In the brain, matured and quiescent astrocytes become reactive after injury, and reactive astrocytes form a barrier around the injured neural area to prevent further lesions, which however, may also lead to inhibition of axonal growth and functional damage^74^. Similarly, activated astrocytes play a major role in the remodelling of the extracellular matrix in optic neuropathy, such as glaucoma, leading to axonal loss and retinal ganglion cell degeneration^75,76^. Therefore, controlling glial activity by modulating neurotrophic factors may have a potential to protect neurons and minimize damage^77–79^. Neurotrophic factors, such as BDNF have been reported to result in delayed activation of the microglial cells and increased RGC survival following optic nerve transection^78^. Modulation of NGF receptors by inhibition of p75^NTR^ (expressed by Muller glia) was also shown to enhance TrkA-mediated survival of injured RGCs^79^, indicating that targeting microglial activation and modulating neurotrophic pathway may be a potential strategy to save retinal neurons following injury. Similarly, our results also show that topical rh-NGF significantly inhibits pONT induced increase in astrocyte activity (GFAP intensity) in the optic nerve, which was consistent with increased axonal survival with higher dose of rh-NGF.

In conclusion, we have demonstrated that topical NGF is neuroprotective to RGCs and their axons after optic nerve injury. The protective effects of NGF are mainly attributed to targeting secondary degeneration. DARC imaging is a useful tool for monitoring NGF efficacy on RGC protection in real time, with implications of translating the therapy to clinic.

## Methods

### Animals

Adult male Dark Agouti rats weighing 150–200 grams were used in this study and treated with procedures approved by the UK Home Office and in compliance with the ARVO Statement for the Use of Animals in Ophthalmic and Vision Research. Animals (n = 46) were maintained in a 12-h light/12-h dark cycle with a room illuminance of 140–260 lux during the bright portion of the cycle, and provided standard food and water *ad libitum*.

### Partial optic nerve transection (pONT)

Under general anaesthesia (GA), pONT was performed in the left eye of all animals, as previously described^7,30^. Briefly, an incision was made in the superior conjunctiva, and the optic nerve sheath exposed. A longitudinal slit was made in the dura mater and a 0.2-mm cross cut performed in the dorsal optic nerve at a distance of 2 mm behind the eye. An ophthalmic scalpel with a steel cutting guard of 0.2-mm was used in this procedure. Damage of major ophthalmic blood vessels was avoided and verified at the end of surgery by ophthalmoscopy.

### rh-NGF topical treatment

Following pONT procedure, animals were topically treated with rh-NGF in two concentrations of 180 ug/ml and 540 ug/ml, respectively, twice a day for 3 weeks. Control groups included pONT without rh-NGF treatment and normal naïve animals. At least 5 animals were included in each treatment and control groups. Researchers were masked to the treatment groups. To assess whether rh-NGF reaches the retina and optic nerve after topical administration, six animals were topically treated with either hr-NGF or vehicle, twice a day for 3 days, and culled 6 hours after the last eye drops.

### DARC RGC apoptosis imaging

All animals were imaged *in vivo* for RGC apoptosis at baseline and 3 weeks after pONT and treatments using DARC, as we previously described^62,65,66^. Briefly, animals under GA were intravitreally administered with fluorescently labelled 488 annexin V and imaged in 2 hours using a confocal scanning laser ophthalmoscopy (cSLO) (HRA Spectralis, Heidelberg Engineering, Heidelberg, Germany). The retinal images were then collected, and the number of apoptotic RGCs were manually counted by three masked observers and the normalised DARC count determined using equation [1].

1 $$D{C}_{ \% }=100\ast \frac{DC-\overline{D{C}_{C}}}{\overline{D{C}_{P}}-\overline{D{C}_{C}}}$$

where DC_%_ is the percentage DARC count, which comprises an individual DARC count (DC) normalised to mean pONT DARC count (DC_p_) and mean naïve control DARC count (DC_c_,).

### Immunohistochemistry and confocal microscopy

Animals were sacrificed after 3 weeks’ *in vivo* study, and both eyes enucleated and fixed in fresh 4% paraformaldehyde at 4 °C overnight. Retinal whole-mounts and optic nerves were then dissected for immunostaining. To assess the therapeutic effects of rh-NGF on RGC protection, retinal whole-mounts were stained with an anti-mouse Brn-3a antibody (1:350, Merck Millipore, MAB1585) and examined under confocal microscopy (LSM 710, Carl Zeiss Microlmaging, GmbH, Jena, Germany). A single plane maximum intensity projection of RGC layer was then obtained following z-stack and tile scan of each retina. Optic nerves were paraffin-embedded and cross-sections obtained before immunostaining with antibodies of anti-mouse neurofilament-H (NF, 1:150, Biolegend, SMI31) and anti-rabbit glial fibrillary acidic protein (GFAP, 1:1000, Dako, Z0334), respectively. The stained optic nerve sections were also examined under confocal microscopy and quantified by fluorescent intensity using Image J.

### Automated quantification of Brn3a labelled RGCs

Quantification of Brn3a labelled RGCs in retinal whole-mounts was achieved using an algorithm previously described^30,31^. Briefly, a high-pass filter was applied to the 8-bit Brn3a labelled channel to remove background followed by application of a 130 intensity threshold. The ImageJ watershed algorithm was then used to separate touching particles and those within 7–21 µm size range were counted based on RGC sizes previously reported^48^, and only particles with a circularity >0.7 were considered to be RGCs in order to exclude blood vessels also stained by Brn3a. Once identified, a region of interest (ROI) was defined for each RGC from which RGC area, mean grey pixel intensity and centroid (x,y) was recorded. The density of RGCs (cells/mm^2^) was calculated by the algorithm counts divided by retinal area which was determined by applying a customised ImageJ script. The nearest neighbour distance (NND) was determined for each RGC using an ImageJ macro from which regularity index (RI) was calculated. To assess topical rh-NGF effects on primary and secondary degeneration, retinal whole-mounts were segmented into quadrants and RGC loss in superior and inferior segments evaluated^31^.

### Human NGF detection in the retina and optic nerve by ELISA

Six animals were sacrificed 3 days following unilateral pONT and topical treatment of either rh-NGF or vehicle. The eyes were enucleated, and the retina and the optic nerves freshly dissected on ice and stored in clean sterile tubes at −80 °C until use. To extract proteins, the retina and optic nerve samples were homogenized by bath sonication in Cell Lysis Buffer (C3228-500 ml CellLytic MT Cell Lysis Reagent, Sigma) containing protease inhibitor cocktail (P8340 Sigma) and phosphatase inhibitor cocktails 2 and 3 (P5726-1ML and P0044-1ML, Sigma). Then, protein lysates were kept in a cold room on a rotating shaker for 2 h to allow complete tissue disaggregation and cell lysis. This step was followed by sample centrifugation at 10,000 × *g* for 20 min at 4 °C. The supernatants were withdrawn to be used to detect human beta-NGF concentration in the retina and optic nerve using a rat beta-NGF human SimpleStep ELISA kit ab193760 (abcam), following the manufacturer’s instructions. Briefly, a SimpleStep ELISA kit uses an affinity tag labelled capture antibody and a reporter conjugated detector antibody which immunocapture the sample analyte in solution. This entire complex (capture antibody/analyte/detector antibody) is, in turn, immobilized via immunoaffinity of an anti-tag antibody coating the well. To perform the assay, 50 µl volume of samples (duplicate) or standards (triplicate) were added to the wells, followed by 50 µl volume of the antibody mix. After 1 h incubation at room temperature on a plate shaker set to 400 rpm, the wells were washed 3 × 350 μL 1X wash buffer to remove unbound material. Then, 100 μL of TMB Development Solution was added to each well followed by an incubation for 5–10 min in the dark (avoiding standard signal saturation) on a plate shaker set to 400 rpm. During incubation, catalyzed by HRP, a blue coloration was generated. This reaction was stopped by addition of 100 μL of Stop Solution to each well, completing any colour change from blue to yellow. Signal was generated proportionally to the amount of bound analyte and the intensity was measured at 450 nm with a plate reader after 1 min on a plate shaker to mix.

### Statistical analysis

All data were analysed with Student t-test or one-way ANOVA with Bonferoni post-test versus control groups using GraphPad Prism 5 (GraphPad Software, Inc., La Jolla, CA, USA). Data were presented as means ± SEM and P < 0.05 was considered significant. N ≥ 5.

## Supplementary information

Supplementary Table 1.

## Data Availability

The datasets generated during and/or analysed during the current study are available from the corresponding author on reasonable request.
