# Supplementary material for: Topical recombinant human Nerve growth factor (rh-NGF) is neuroprotective to retinal ganglion cells by targeting secondary degeneration
Source: Sci Rep. 2020 Feb 25;10:3375. doi: 10.1038/s41598-020-60427-2 (PMC7042238; doi:10.1038/s41598-020-60427-2)
Supplement: Supplementary file 1 — Supplementary Table 1. [file 41598_2020_60427_MOESM1_ESM.pdf]

Topical recombinant human Nerve growth factor (rh-NGF) is neuroprotective to retinal ganglion cells by targeting secondary degeneration

Li Guo,[1] Benjamin M. Davis,[1] Nivedita Ravindran,[1] Joana Galvao,[1] Neel Kapoor,[1] Nasrin Haamedi,[1] Ehtesham Shamsher,[1] Vy Luong,[1] Elena Fico[1,2] and M. Francesca Cordeiro[1,3 *]*

[1]Glaucoma & Retinal Neurodegeneration Research Group, Institute of Ophthalmology, University College London, London, United Kingdom; [2] Department of Biotechnological and Applied Clinical Sciences, University of L'Aquila, Italy; [3] Western Eye Hospital, Imperial College Healthcare NHS Trust, London, United Kingdom

Supplementary table 1 Qualitative assessment of radioactivity in the ocular tissues of male albino rats after the final topical administration of [^{3}H]-rh-NGF at a nominal dose level of 1.0 µg/eye

**Animal identity**	179		183		185		187		189	
**Eyes**	Left	Right	Left	Right	Left	Right	Left	Right	Left	Right
**Tissue & Sampling time**	2 hours		4 hours		8 hours		12 hours		24 hours	
**Lens[1]**	+++	+++	+++	+++	+++	NS	+++	+++	+++	+++
**Cornea**	++	++	++	++	++	NS	++	++	++	++
**Ciliary body**	+	+	+	+	+	NS	+	+	+	+
**Iris**	+	+	+	+	+	NS	+	+	+	+
**Choroid**	+	+	+	+	+	+	+	+	+	+
**Retina**	+	+	+	+	+	+	+	+	+	+
**Sclera**	++	++	++	++	++	++	++	++	++	++
**Optic nerve**	+	+	+	+	+	+	+	+	+	+
**Aqueous humour**	BGL	BGL	BGL	BGL	BGL	BGL	BGL	BGL	BGL	BGL
**Vitreous humour**	BGL	BGL	BGL	BGL	BGL	BGL	BGL	BGL	BGL	BGL

Silver grains detected in the ocular tissues at high (+++), moderate (++), and low (+) levels, and background levels (BGL). Lens[1]: elevated levels found in the lens compared to quantitative assessment, suggesting pressure artefacts caused by creasing/founding of the tissue against the nuclear emulsion. NS: Tissue not sectioned. (The table results were kindly provided by the manufacture, Dompe).
